# Association between Leptin and Complement in Hepatitis C Patients with Viral Clearance: Homeostasis of Metabolism and Immunity

**DOI:** 10.1371/journal.pone.0166712

**Published:** 2016-11-21

**Authors:** Ming-Ling Chang, Chia-Jung Kuo, Hsin-Chih Huang, Yin-Yi Chu, Cheng-Tang Chiu

**Affiliations:** 1 Liver Research Center, Division of Hepatology, Department of Gastroenterology and Hepatology, Chang Gung Memorial Hospital, Taoyuan, Taiwan; 2 Department of Medicine, College of Medicine, Chang Gung University, Taoyuan, Taiwan; Università degli Studi di Palermo, ITALY

## Abstract

**Background:**

The association between leptin and complement in hepatitis C virus (HCV) infection remains unknown.

**Methods:**

A prospective study was conducted including 474 (250 genotype 1, 224 genotype 2) consecutive chronic hepatitis C (CHC) patients who had completed an anti-HCV therapy course and undergone pre-therapy and 24-week post-therapy assessments of interferon λ3-rs12979860 and HCV RNA/genotypes, anthropometric measurements, metabolic and liver profiles, and complement component 3 (C3), C4, and leptin levels.

**Results:**

Of the 474 patients, 395 had a sustained virological response (SVR). Pre-therapy leptin levels did not differ between patients with and without an SVR. Univariate and multivariate analyses showed that sex (pre- and post-therapy, p<0.001), body mass index (BMI) (pre- and post-therapy, p<0.001), and C3 levels (pre-therapy, p = 0.027; post-therapy, p = 0.02) were independently associated with leptin levels with or without HCV infection. Pre-therapy BMI, total cholesterol (TC), C4 levels, and the rs12979860 genotype were independently associated with pre-therapy C3 levels in all patients. Post-therapy BMI, alanine aminotransferase, TC, C4 levels, white blood cell counts, and hepatic steatosis were independently associated with the post-therapy C3 levels of SVR patients. Compared with pre-therapy levels, SVR patients showed higher 24-week post-therapy C4 (20.32+/-7.30 vs. 21.55+/-7.07 mg/dL, p<0.001) and TC (171.68+/-32.67 vs. 186.97+/-36.09 mg/dL, p<0.001) levels; however, leptin and C3 levels remained unchanged after therapy in patients with and without an SVR.

**Conclusions:**

Leptin and C3 may maintain immune and metabolic homeostasis through association with C4 and TC. Positive alterations in C4 and TC levels reflect viral clearance after therapy in CHC patients.

## Introduction

Hepatitis C virus (HCV), a human pathogen responsible for acute and chronic liver disease, has variants classified into 7 major genotypes and infects an estimated 170 million individuals worldwide [1]. It affects insulin signaling, and much of its life cycle is closely associated with lipid metabolism [2]. In addition to cirrhosis and hepatocellular carcinoma, HCV is thought to cause metabolic alterations, including steatosis, dyslipidemia, insulin resistance (IR), diabetes, obesity, and cardiovascular events [2–4]. Although most HCV infections are currently curable using potent direct-acting anti-viral agents, not all HCV-associated cardiometabolic complications are reversible after viral clearance [2]. Adipose tissue has emerged as an important endocrine organ that exerts vital endocrine and immune functions via adipokines [5]. Moreover, free fatty acids and glycerol derived from visceral adipose tissue reach the liver and stimulate the biosynthesis of lipoprotein and glucose, respectively [6]. Because adipose tissues and the liver are functionally linked, elucidating the relationship between adipokine alterations and HCV infection has the potential to reveal the basis of HCV-associated cardiometabolic complications and probe the therapeutic targets.

The adipokine leptin, a product of the obese gene, is primarily expressed in adipose tissue but is also expressed in other organs, including the liver [7]. Most of the circulating leptin originates from subcutaneous, but not visceral adipose tissue, which may reduce its biological activity [5]. Leptin is crucial for maintaining total body fat and glucose homeostasis as well as regulating food intake and energy expenditure through a complex central feedback mechanism [8]. Its secretion is influenced by numerous physiological and hormonal factors. The leptin receptor is expressed in hypothalamic neurons, T cells, and hepatic stellate cells [9]. Importantly, leptin promotes IR to increase intracellular fatty acids in hepatocytes, amplifies proinflammatory responses [10], and mediates hepatic fibrogenesis during chronic liver injury [11] through the activation of hepatic stellate cells [12]. Concordantly, leptin levels are elevated in patients with a higher fibrosis index [13]. Importantly, leptin is critical for the modulation of adaptive and innate immune responses, such as regulating T-cell-mediated immune responses [14] and natural killer cell activity [15], as well as increasing complement component 3 (C3) levels [16]. Because both HCV infection and leptin are critically involved in metabolism, inflammation, and immunity [5–16], their potential relationship has attracted attention; however, no definite conclusion regarding such a relationship has been drawn [16–21]. In addition to the multifaceted functions of leptin, this uncertainty is primarily due to variability among individuals, which is difficult to completely eliminate from case-control studies, retrospective studies, or prospective studies with inadequate confounder adjustments. Indeed, although the impact of HCV infection on alterations in leptin levels is unclear, even less is known regarding whether viral genotype-specific influences on these alterations exist [21–23]. Therefore, we sought to elucidate the aforementioned relationships by conducting a prospective study to analyze the leptin levels adjusting for viral, metabolic, and immune profiles in genotype 1 (G1) and genotype 2 (G2) CHC patients who completed anti-HCV therapy.

## Methods

### Patients

The study group comprised subjects 18 years or older with G1 or G2 CHC, which was defined as the presence of documented HCV antibody positivity and detectable HCV RNA for >24 weeks. Subjects with heavy alcohol consumption (alcohol consumption more than 10 g/day for women and 20 g/day for men [5]), human immunodeficiency virus infection, hepatitis B infection, hemochromatosis, coronary heart disease, renal insufficiency, or malignancy and recipients of solid organ transplants were excluded.

### Methods

A total of 250 G1 and 224 G2 CHC patients were consecutively recruited at a tertiary referral center between July 2010 and June 2015. All patients received anti-HCV therapy with weight-based pegylated interferon-α-2b (1.5 μg/kg/week) and ribavirin (800–1400 mg/day) for either 24 or 48 weeks according to the therapeutic response-guided protocol [1,4]. The HCV RNA levels were assessed using a COBAS Amplicor (Roche Diagnostics, Tokyo, Japan). The HCV genotype was determined using the InoLipa method (Roche Diagnostics). Single nucleotide polymorphisms of interferon λ3 (IFNL3 or interleukin-28B) rs12979860 were assessed using genomic DNA, as previously described [1,4]. The patients were evaluated for HCV RNA to examine the therapeutic response 2 weeks prior to therapy, after 4, 12, and 24 weeks of therapy, at the end of therapy, and 12 and 24 weeks after the end of therapy. At 2 weeks prior to therapy and 24 weeks after the end of therapy, after fasting, the patients were evaluated for body mass index (BMI), total cholesterol (TC), high-density lipoprotein-cholesterol (HDL-C), triglycerides (TGs), uric acid, homeostasis model assessment-estimated insulin resistance (HOMA-IR) [fasting insulin (μU/mL) × fasting glucose (mmol/L)/22.5], alanine aminotransferase (ALT), aspartate aminotransferase (AST)-to-platelet ratio index (APRI), white blood cell (WBC) count, platelet count, and high-sensitivity C-reactive protein (hsCRP), C3, C4, and leptin (R&D Systems, MN, USA) levels. Abdominal ultrasound studies were performed in every patient prior to therapy and every 6 months thereafter to assess the presence and severity of fatty liver and cirrhosis. IR was defined as a HOMA-IR score ≥2.5. A sustained virological response (SVR) was defined as undetectable HCV RNA levels 24 weeks after the completion of therapy.

A liver biopsy was performed in every patient before anti-HCV therapy to assess the liver histology. The liver biopsy specimens were semi-quantitatively scored by an experienced hepatopathologist blinded to the clinical data. Histological scores for steatosis and fibrosis were reported using the criteria of Kleiner et al. [24] and staged according to the Metavir scoring system [25], respectively.

### Statistics

All statistical analyses were performed using the Statistical Package for Social Science software (SPSS ver. 21.0, SPSS Inc., Chicago, IL, USA). Continuous variables were analyzed using Student’s t-test or the Mann-Whitney U test, and categorical variables were analyzed using the chi-square test or Fisher’s exact test, as appropriate. Univariate and multivariate linear regression models were used to assess the relationships between various pre-therapy dependent and independent variables. Paired t-tests were used to compare the variables prior to and 24 weeks after anti-HCV therapy within the same individuals. The variable values were logarithmically transformed and then used for the statistical analyses where indicated. Statistical significance was defined at the 5% level based on two-tailed tests of the null hypothesis.

### Informed consent

Written informed consent was obtained from each patient. The study protocol conformed to the ethical guidelines of the 1975 Declaration of Helsinki and was approved by the Chang Gung Memorial Hospital institutional review board.

## Results

### Pre-therapy leptin levels did not differ between patients with and without an SVR

The baseline characteristics of the 474 CHC patients are listed in Table 1. Patients with an SVR (n = 395) had a lower BMI, lower HCV RNA and HOMA-IR levels, and a lower prevalence of G1 HCV infection and severe fibrosis (F3 and F4) but a higher prevalence of the IFNL3-rs12979860 CC genotype than patients without an SVR (n = 79). No differences in pre-therapy leptin levels were noted between the patients with and without an SVR. With regard to the genotype impact on leptin, no difference in the pre-therapy leptin levels was noted between the G1 and G2 patients after sex stratification (p = 0.328 for the males and p = 0.177 for the females).

**Table 1:** Baseline characteristics of CHC patients.

	All CHC patients (n = 474)	Patients with SVR (n = 395)	Patients without SVR (n = 79)	Student’s *t*-test p-values
Male, n (%)	270 (57.0)	228 (57.7)	42 (53.2)	0.456
Age (yr)	55.18+/-11.92	53.72+/-11.61	55.99+/-10.59	0.109
BMI	24.89+/-3.78	24.79+/-3.59	25.77+/-4.10	0.035*
HCV RNA (Log_10_IU/ml)	5.97+/-1.12	5.84+/-1.18	6.45+/-0.75	<0.001*
HCV genotype [G1, n (%)]	250 (52.7)	187 (47.3)	63 (79.7)	<0.001*
AST (U/L)	75.06+/-68.55	73.06+/-61.63	73.74+/-63.90	0.933
ALT (U/L)	92.20+/-94.24	97.53+/-88.50	80.39+/-64.12	0.124
TC (mg/dL)	171.55+/-34.55	171.7+/-32.7	172.7+/-29.8	0.805
HDL (mg/dL)	42.82+/-13.81	48.22+/- 13.80	43.12+/-13.8	0.867
TG (mg/dL)	104.85+/-54.96	106.19+/-57.88	96.13+/-39.4	0.099
HOMA-IR	3.21+/-5.38	2.88+/-3.03	4.46 +/-9.42	0.039*
Uric acid (mg/dL)	5.91+/-1.56	5.94+/-1.54	5.86+/-1.50	0.709
WBC count (10^3^/μL)	5.72+/-3.23	5.82+/-1.91	5.63+/-1.73	0.433
Platelets (10^3^/μL)	176.3+/-64.3	181.4+/-58.1	156.0+/-58.7	0.001*
hsCRP (mg/dL)	1.87+/-3.72	1.68+/-3.13	1.56+/-1.95	0.774
C3 (mg/dL)	105.80+/-20.00	107.4+/-19.8	102.6+/-17.2	0.088
C4 (mg/dL)	20.42+/-7.89	20.56+/-7.70	19.25+/-7.28	0.238
APRI	1.65+/-2.05	1.47+/-1.62	1.75+/-1.83	0.195
Hepatic steatosis				
None, n (%)	166 (35)	150 (33)	16 (20.2)	0.395
Mild, n (%)	213 (45)	176 (44.6)	37 (46.8)	0.5
Moderate, n (%)	85 (17.9)	62 (15.7)	23 (29.1)	0.49
Severe, n (%)	10 (2.1)	7 (2.5)	3 (3.7)	0.48
Fibrosis				
F0, n (%)	76 (16)	68 (17.2)	8 (10.1)	0.095
F1, n (%)	171 (36)	151 (38.2)	20 (25.3)	0.17
F2, n (%)	166 (35)	138 (34.9)	28 (35.4)	0.317
F3, n (%)	38 (8)	25 (6.3)	13 (16.4)	0.049*
F4, n (%)	23 (5)	13 (3.3)	10 (12.6)	0.032*
Leptin (pg/ml)	9748.6+/-9968.2	9332+/-9049	12168+/-14092	0.267
Log leptin (Log_10_pg/ml)	3.77+/-0.52	3.75+/-0.54	3.83+/-0.48	0.4
SNP rs12979860 CC, n (%)	401 (85)	348 (88.1)	53 (67.1)	0.013*

BMI: body mass index; G1: genotype 1; AST: aspartate aminotransferase; ALT: alanine aminotransferase; TC: total cholesterol; HDL: high-density lipoprotein cholesterol; TGs: triglycerides; HOMA-IR: homeostasis model assessment-estimated insulin resistance; WBC: white blood cell; hsCRP: high-sensitivity C-reactive protein; C3: complement component 3; C4: complement component 4; APRI: AST to platelet ratio index; Log: logarithmic; SNP: single nucleotide polymorphism;

*: *p*<0.05.

### Sex, BMI, and C3 levels were independently associated with leptin levels regardless of viral presence

The results of the univariate and multivariate analyses performed to determine the factors associated with the pre-therapy (for all patients, n = 474) and 24-week post-therapy (for patients with an SVR, n = 395) leptin levels are listed in Table 2. The univariate analyses revealed that sex, pre-therapy BMI, C3 levels, and hepatic steatosis were associated with pre-therapy leptin levels, whereas the multivariate analysis showed that sex, pre-therapy BMI, and C3 levels were independently associated with the pre-therapy leptin levels. Regarding the post-therapy leptin levels, the univariate analyses showed an association with sex, post-therapy BMI, HOMA-IR, C3 levels, and hepatic steatosis, whereas the multivariate analyses showed that sex, post-therapy BMI, and C3 levels were associated factors. Regarding the pre-therapy HCV RNA levels, age [estimated β: -0.054, 95% confidence interval (CI) of β: -0.105–0.004, p = 0.035] and pre-therapy TGs (estimated β: 0.021, 95% CI of β: 0.01–0.033, p<0.001) were independent factors.

**Table 2:** Univariate and multivariate analyses of factors associated with pre- and post-therapy leptin levels.

	Pre-therapy leptin (all patients)		Post-therapy leptin (patients with SVR)	
	Univariate analysis 95% CI of β [β](p value)	Multivariate analysis 95% CI of β [β](p value)	Univariate analysis 95% CI of β [β](p value)	Multivariate analysis 95% CI of β [β](p value)
Sex (male)	-11325~-6839[–8857](<0.001*)	-12362.7~-8313.0[-10333.7](<0.001*)	-13016~-7235[–10126](<0.001*)	-39774.2~-18863.2[-12592.2](<0.001*)
Age (yr)	-108.9~138.9[11.48] (0.851)		-88.2~204.0[57.8](0.436)	
BMI	999.7~1649.7 [1324](<0.001*)	1075.7~1679.3 [1377.5](<0.001*)	729.3~1226.4[1210] (<0.001*)	1013.8~1813.8[1422.8] (<0.001*)
HCV RNA (Log_10_IU/ml)	-493.8~2189.0[847.6](0.214)		NA	
HCV genotype	-2133.3~456.3[-838.4] (0.203)		NA	
AST (U/L)	-26.3~16.5[-4.81](0.653)		-227.0~109.2[-58.9](0.49)	
ALT (U/L)	-22.8~8.8[-7.0](0.384)		-94.1~115.9[10.9](0.838)	
TC (mg/dL)	-34.9~49.0[7.04](0.749)		-40.5~48.9[4.17](0.854)	
HDL (mg/dL)	-115.7~96.4[-9.6](0.858)		-15.8~233.8 [109.0](0.087)	
TG (mg/dL)	-10.1~43.3[16.6](0.223)		-21.4~25.8[2.22](0.853)	
HOMA-IR	-108.7~348.7[119.9](0.302)		32.8~935.6[484.2](0.036*)	-216.0~419.6[126.8](0.466)
Uric acid (mg/dL)	-668.1~1113.9[222.9](0.622)		-1677.9~346.3[-665.8](0.196)	
WBC count (10^3^/μL)	-459.3~941.0[241.0](0.498)		-997.9~748.1[-124.9](0.778)	
Platelets (10^3^/μL)	-12.1~32.8[10.37](0.364)		-45.8~11.6[-17.1](0.242)	
hsCRP (mg/dL)	-1.7~987.5[492.8](0.051)		-148.9~444.6[147.8](0.327)	
C3 (mg/dL)	72.1~207.5[139.8](<0.001*)	7.2~116.3[61.7](0.027*)	140.2~330.5[236.4](<0.001*)	16.8~185.8[101.0](0.02*)
C4 (mg/dL)	-120.1~240.2[59.1](0.521)		-36.6~435.2[199.4](0.097)	
APRI	-1183.1~348.7[-417.2](0.284)		-6173.4~4671.5[-740.8](0.788)	
Hepatic steatosis (yes)	2191.3~7591.7[4879](<0.001*)	-1527.6~2820.2[646.3] (0.558)	2275.8~8578.8[5420.5](0.001*)	-501.9~4558.3 [2028.2](0.115)
SNP rs12979860	-2359.9~4020.2[830.1](0.608)		-2893.1~4819.8[963.4] (0.622)	

BMI: body mass index; AST: aspartate aminotransferase; ALT: alanine aminotransferase; TC: total cholesterol; HDL: high-density lipoprotein cholesterol; TGs: triglycerides; HOMA-IR: homeostasis model assessment-estimated insulin resistance; WBC: white blood cell; hsCRP: high-sensitivity C-reactive protein; C3: complement component 3; C4: complement component 4; APRI: AST to platelet ratio index; SNP: single nucleotide polymorphism;

*: *p*<0.05.

Because the HOMA-IR was reported to be positively correlated with the leptin serum levels in nondiabetic CHC patients [19,26], we stratified the patients by the presence of IR and performed univariate and multivariate analyses to determine the leptin levels. Pre-therapy HOMA-IR was an independent factor for pre-therapy leptin in patients without IR (estimated β: 3498, 95% CI: 1395–5600, p = 0.001) but not in patients with IR (p = 0.521).

### TC and C4 levels were independently associated with C3 levels regardless of viral presence

**Fig 1:**
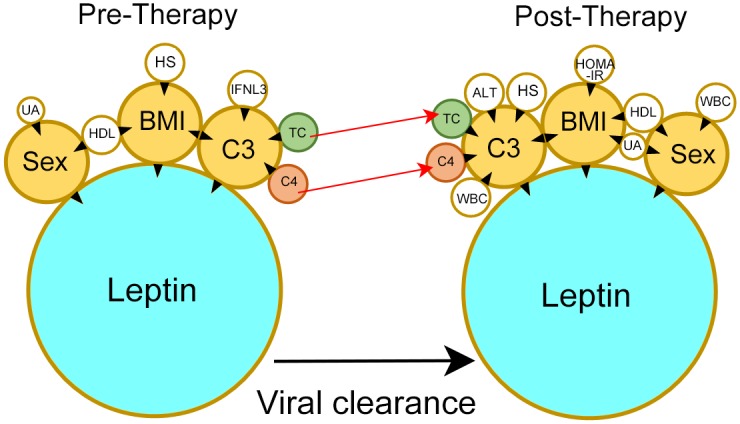
The leptin-centered associations between the dependent and independent factors before (pre-therapy) and 24 weeks after anti-hepatitis C therapy (post-therapy). Tips of black arrowheads: dependent factors; Bases of black arrowheads: independent factors; UA: uric acid; HDL-C: high-density lipoprotein-cholesterol; BMI: body mass index; HS: hepatic steatosis; IFNL3: interferon, λ3; TC: total cholesterol; C3: complement component 3; complement component 4: C4; WBC: white blood cell; HOMA-IR: homeostasis model assessment-estimated insulin resistance; red arrows: post-therapeutic increases in the TC and C4 levels.

Because sex, BMI, and C3 levels were associated with the leptin levels regardless of the presence of HCV, we investigated the factors associated with sex, BMI, and C3 using univariate and multivariate analyses (Table 3). Regarding the pre-therapy levels of all the patients, the univariate and multivariate analyses showed that uric acid and HDL-C were independently associated with sex; HDL-C, C3, and hepatic steatosis were independently associated with BMI; and BMI, TC, C4, and the IFNL3-rs12979860 CC genotype were independently associated with the C3 level. Regarding the post-therapy levels of the patients with an SVR, HDL-C, uric acid, and WBC count were independently associated with sex; HDL, HOMA-IR, uric acid, and C3 were independently associated with BMI; and BMI, ALT, TC, WBC count, hepatic steatosis, and C4 were independently associated with the C3 level. The associated leptin-centered relationships between the dependent and independent factors (pre-therapy and post-therapy) are summarized in Fig 1.

**Table 3:** Multivariate analyses of factors associated with pre- and post-therapy sex, BMI, and C3 levels.

	Pre-therapy dependent variables (all patients)			Post-therapy dependent variables (SVR patients)		
	Sex	BMI	C3	Sex	BMI	C3
	Multivariate analysis, 95% CI of OD [OD], (p value)	Multivariate analysis, 95% CI of β [estimated β], (p value)		Multivariate analysis, 95% CI of OD [OD], (p value)	Multivariate analysis, 95% CI of β [estimated β], (p value)	
Sex (male)					-0.701~1.075 [0.187](0. 679)	
BMI			0.441~1.514 [0.978](<0.001*)	0.893 ~1.070 [0.977](0.621)		0.35~1.318 [0.834](0. 001*)
ALT (U/L)	1.0~1.004[1.002](0.0059)		0.0~0.06 [0.03](0.05)	0.987~1.025 [1.006] (0.529)	-0.01~0.04 [0.015](0.239)	0.092~0.296 [0.194] (<0.001*)
TC (mg/dL)	0.914~1.005 [1.0] (0.934)		0.046~0.184 [0.115](0.001*)			0.027~0.019 [0.073](0.002*)
HDL (mg/dL)	0.934~0.965 [0.949](<0.001*)	-0.077~-0.018 [-0.048](0.002*)	-0.295~0.074 [-0.11](0.24)	0.903~0.952 [0.927] (<0.001*)	-0.08~-0.011 [-0.046](0.009*)	-0.18~0.099 [-0.04](0.568)
TG (mg/dL)		-0.009~0.007 [-0.001](0. 778)	-0.006~0.085 [0.039](0.087)		-0.009~0.002 [-0.003](0.271)	-0.023~0.025 [0.001](0.936)
HOMA-IR		-0.018~0.137 [0.059](0.131)	-0.344~0.385 [0.02](0.912)		0.077~0.315 [0.196](0.001*)	-0.102~0.898 [0.398](0.118)
Uric acid (mg/dL)	1.462~1.919 [1.675] (<0.001*)		-0.443~2.305[0.931](0.183)	1.41~2.233 [1.775] (<0.001*)	0.188~0.745 [0.466](0.001*)	-1.128~1.090 [-0.019](0.973)
WBC count (10^3^/μL)	0.975~1.184 [1.074](0.149)	-0.05~0.346 [0.148](0.141)	-0.779~1.452[0.337](0.553)	1.011~1.411 [1.194](0.037*)	-0.156~0.33 [0.087](0.481)	0.285~2.269 [1.277](0.012*)
Platelets (10^3^/μL)			-0.028~0.061 [0.017](0.462)		-0.003~0.012 [0.004](0.277)	-0.013~0.049 [0.018](0.265)
hsCRP (mg/dL)		-0.082~0.208 [0.063] (0.39)	-0.261~0.188 [0.463](0.209)		-0.041~0.114[0.037](0.355)	-0.037~0.606 [0.285](0.082)
C3 (mg/dL)		0.028~0.073 [0.05](<0.001*)			0.019~0.075 [0.047](0.001*)	
C4 (mg/dL)	0.987~1.034 [0.558](0.383)	-0.051~0.064 [0.007](0.815)	0.74~1.302 [1.021](<0.001*)		-0.047~0.064[0.008](0.766)	0.512~0.943 [0.728](<0.001*)
APRI			-2.231~1.415 [-0.408](0.66)			
Hepatic steatosis (yes)		0.354~1.885 [1.12](0.004*)	-2.4~5.7 [1.654](0.423)		-0.47~0.064 [0.298] (0.445)	0.341~6.651 [3.496](0.03*)
SNP rs12979860 CC genotype			1.233~9.747 [5.49](0.012*)			

Only the variables significant in the univariate analyses (data not shown) were included in the multivariate analyses; OR: odds ratio; BMI: body mass index; AST: aspartate aminotransferase; ALT: alanine aminotransferase; TC: total cholesterol; HDL: high-density lipoprotein cholesterol; TGs: triglycerides; HOMA-IR: homeostasis model assessment-estimated insulin resistance; WBC: white blood cell; hsCRP: high-sensitivity C-reactive protein; C3: complement component 3; C4: complement component 4; APRI: AST to platelet ratio index; SNP: single nucleotide polymorphism

*: *p*<0.05.

### After anti-HCV therapy, the lipid profile and C4 levels increased, and the C3 and leptin levels remained unchanged

Although trivial, patients with and without an SVR had a decreased BMI after anti-HCV therapy. However, only patients with an SVR had decreased AST, ALT, and APRI levels but increased lipid profiles, uric acid, and C4 levels. The leptin and C3 levels remained unchanged regardless of the therapeutic response (Table 4).

**Table 4:** Comparison of the pre- and 24-week post-therapy variables in CHC patients stratified by the therapeutic response.

	SVR (+) (*n* = 395)		Paired *t*-test *p*-values	SVR (-) (*n* = 79)		Paired *t*-test *p*-values
	Pre-therapy value	Post-therapy value		Pre-therapy value	Post-therapy value	
BMI	24.77+/-3.65	24.36+/-3.53	<0.001*	25.84+/-4.10	24.86+/-3.60	<0.001*
AST (U/L)	73.08+/-62.01	26.21+/-11.15	<0.001*	74.93+/-64.40	73.37+/-66.06	0.841
ALT (U/L)	97.40+/-88.67	21.64+/-14.70	<0.001*	81.60+/-64.52	71.20+/-67.80	0.799
TC (mg/dL)	171.68+/-32.67	186.97+/-36.09	<0.001*	173.54+/-29.79	171.85+/-32.81	0.615
HDL (mg/dL)	48.15+/-13.80	49.85+/-13.35	<0.001*	48.83+/-14.82	49.91+/-14.77	0.314
TG (mg/dL)	101.35+/-46.74	120.73+/-75.58	<0.001*	115.97+/-66.98	104.79+/-47.70	0.069
HOMA-IR	2.88+/-4.75	2.74+/-2.98	0.374	4.50+/-5.94	4.69+/-7.81	0.703
Uric acid (mg/dL)	5.89+/-1.52	6.13+/-1.54	<0.001*	5.88+/-1.48	5.89+/-1.46	0.9
WBC count (10^3^/μL)	5.85+/-1.94	5.83+/-1.81	0.801	5.56+/-1.69	5.13+/-1.26	0.032*
Platelets (10^3^/μL)	182.3+/-58.78	184.53+/-56.51	0.239	154.9+/-58.73	148.9+/-54.2	0.203
hsCRP (mg/dL)	1.59+/-2.73	1.78+/-4.46	0.494	1.58+/-1.99	1.90+/-3.64	0.436
C3 (mg/dL)	107.2+/-19.74	108.6+/-17.39	0.169	101.9+/-16.66	102.6+/-17.58	0.677
C4 (mg/dL)	20.32+/-7.30	21.55+/-7.07	<0.001*	19.08+/-7.77	19.47+/-8.33	0.536
APRI	1.47+/-1.65	0.50+/-0.39	<0.001*	1.76+/-1.84	1.71+/-1.75	0.845
Hepatic steatosis (yes)	(51)	(52)	0.630	(43)	(49)	0.288
Leptin (pg/mL)	9441.4+/-8992.8	9809.4+/-9448.8	0.543	10229.7+/-10528.5	10821.7+/-12650.4	0.763
Log leptin (Log_10_pg/mL)	3.74+/-0.55	3.77+/-0.54	0.392	3.83+/-0.38	3.85+/-0.40	0.835

SVR: sustained virological response; Log: log transformation; BMI: body mass index; AST: aspartate aminotransferase; ALT: alanine aminotransferase; TC: total cholesterol; HDL: high-density lipoprotein cholesterol; TGs: triglycerides; HOMA-IR: homeostasis model assessment-estimated insulin resistance; WBC: white blood cell; hsCRP: high-sensitivity C-reactive protein; C3: complement component 3; C4: complement component 4; APRI: AST to platelet ratio index; SNP: single nucleotide polymorphism

*: *p*<0.05.

## Discussion

To the best of our knowledge, this prospective study is the first to demonstrate the relationship between leptin and complements in CHC patients. There were several compelling results. (1). No differences in pre-therapy leptin levels were noted between patients with and without an SVR or between G1 and G2 patients. (2). Leptin levels were not associated with HCV RNA levels. (3). Sex, BMI, and C3 levels were independently associated with the leptin levels regardless of the presence of HCV. (4). The IFNL3 genotype, pre-therapy BMI, TC, and C4 were associated with pre-therapy C3 levels, whereas post-therapy BMI, ALT, TC, WBC count, C4, and hepatic steatosis were associated with post-therapy C3 levels. (5). Although both the leptin and C3 levels remained unchanged after viral clearance, C4 and TC, which were independent factors for C3, were significantly increased 24 weeks post-therapy.

Because both fibrosis and the IFNL3 non-CC genotype are two well-documented negative factors for SVR in interferon-based therapy [2], the reliability of this prospective study of 474 CHC patients who had completed a course of anti-HCV therapy is assured by the fact that the non-SVR patients had significantly more advanced fibrosis but a lower prevalence of the IFNL3 CC genotype than the SVR patients. Whether hyperleptinemia is associated with HCV infection [17,18,23] and the pan-genotypic [27,28] or genotype-specific anti-HCV therapeutic responses [29] has remained a matter of debate. In this study, based on the lack of an association between the leptin and HCV RNA levels, and the fact that the leptin levels remained unchanged after an SVR, it is likely that any relationship between leptin alterations and HCV infections is indirect rather than direct. Furthermore, the role of leptin levels in predicting the anti-HCV therapeutic response is negligible, regardless of the HCV genotype, as the pre-therapy leptin levels between the SVR and non-SVR patients and between G1 and G2 patients were similar.

Due to the unavailability of immunoprecipitation which directly assesses the interaction between two proteins [30], it is truly difficult to elucidate the role of leptin in homeostasis upon viral clearance in clinical studies of CHC, especially as the pre- and post-therapy leptin levels were similar. Thus, we adopted the "concept" of popular software programs that organize high-throughput bioinformatic data, such as Metacore and IPA [31], to dissect the interactions between the independent and dependent factors based on the statistical results and the literature. Previous studies of 133~194 nondiabetic CHC patients [19, 26] showed a positive relationship between HOMA-IR and leptin. Consistently, we could only demonstrate this trend in the patients without IR but not in those with IR. All of the above confirmed that the connection between the HOMA-IR and leptin levels vanished with deteriorating glucose metabolism, which may be an HCV-associated sequela [2]. In contrast, sex, BMI, and C3 were independently associated with the leptin level regardless of the presence of HCV infection. With regard to sex and BMI, that female and obese patients have higher leptin levels than male and lean subjects, respectively, is a central dogma of leptin dynamics [5]. The positive association of leptin with C3 is likely due to the regulation of leptin in innate immunity [15] and the co-association of leptin with BMI [5], which was consistent with the results of previous non-HCV studies of the co-culture of adipocytes with macrophages [32] and of obese subjects [16]. The above findings seemed to indicate a non-HCV-specific phenomenon in a CHC cohort. However, the close association between leptin and C3 and the different trends in post-therapeutic changes in C3 and C4 suggest that HCV infection may affect leptin in a qualitative but not quantitative manner through leptin-associated factors. Both C3 and C4 are major proteins of the complement pathways [33], and their synthesis has been shown to be transcriptionally downregulated by the HCV core and NS5A proteins in *in vitro* studies [34,35]. However, this negative regulation is not compatible with the results of either our previous study on conditional HCV core-expressing mice, which demonstrated C3 up-regulation in inflamed liver samples via microarray analyses [36], or with studies of CHC patients who had higher C3 and C4 levels than the controls [37]. The discrepancy may result from the fundamental differences between *in vitro* and *in vivo* immunological studies. In this clinical prospective study, we would like to stress that the positive associations between leptin and C3 and among C3, C4, and TC were consistent regardless of HCV infection (Fig 1). Interestingly, only C4 and TC but not C3 or leptin levels increased after SVR. Additionally, the pre-therapy IFNL3 genotype, a strong determinant of SVR [1,2,4], was associated with the pre-therapy C3 level, although this association diminished after anti-HCV therapy. Instead, the post-therapy C3 levels were affected by the post-therapy WBC count and ALT after viral clearance (Fig 1). This evolution suggests that C3 probably plays a role in HCV clearance. However, the role becomes non-HCV-specific after viral clearance. In contrast to leptin, which is primarily expressed in subcutaneous adipose tissue [5], higher expression levels of C3 and C4 have been reported in visceral than in subcutaneous adipose tissue [38]. The close associations between leptin, C3, and C4 suggest the presence of a strong collaboration between visceral and subcutaneous adipose tissues, which may be essential for maintaining whole-body homeostasis. Collectively, these findings highlight the general importance of leptin in homeostasis, as it needs to remain stable during viral infection but may modulate the immune response through C3, whose levels also remain stable but seem to facilitate immunity and metabolism in conjunction with C4 and TC, respectively. After anti-HCV therapy, only SVR patients had decreased levels of transaminase and APRI but increased lipid profile levels, It is subsequent to the reversal of HCV-associated hepatic injury and hypolipidemia after viral clearance [2,4]. Similarly, the increase in C4 after viral clearance indicated the reversal of the HCV-associated down-regulation of the complement system [34,35].

Because adipose tissue is the major source of leptin [4], the main limitation of this study is the lack of a pathological study of adipose tissue. Moreover, making conclusions based on analyzing the associated factors is an imperfect way to build a complete picture of the leptin-associated pathways. Future studies of leptin in CHC patients with adipose tissue pathology surveys and associated fundamental cellular or animal models studies such as immunopreciptation [30] may be required to elucidate the genuine connection and molecular basis between leptin and C3.

Together, our results demonstrate that sex, BMI, and C3 levels are independently associated with leptin levels and that TC and C4 levels are independently associated with C3 levels regardless of the presence of HCV. Compared with the pre-therapy levels, leptin and C3 remained unchanged 24 weeks post-therapy regardless of the therapeutic response, whereas the levels of C4 and TC increased among patients with an SVR. During HCV infection, leptin and C3 may maintain the homeostasis of metabolism and immunity based on associations with C4 and TC, the positive post-therapy alterations of which reflect viral clearance. These findings will facilitate the development of agents or strategies to probe viral-related metabolic and immunity alterations.

## Supporting Information

S1 Dataset The primary data of the current study are attached as supporting information. (XLS)
